# A hands‐on guide to use network video recorders, internet protocol cameras, and deep learning models for dynamic monitoring of trout and salmon in small streams

**DOI:** 10.1002/ece3.11246

**Published:** 2024-05-27

**Authors:** Konrad Karlsson

**Affiliations:** ^1^ Department of Aquatic Resources, Institute of Freshwater Research Swedish University of Agricultural Sciences Drottningholm Sweden

**Keywords:** AI, CCTV, deep learning models, ecological monitoring, fish, object classification

## Abstract

This study outlines a method for using surveillance cameras and an algorithm that calls a deep learning model to generate video segments featuring salmon and trout in small streams. This automated process greatly reduces the need for human intervention in video surveillance. Furthermore, a comprehensive guide is provided on setting up and configuring surveillance equipment, along with instructions on training a deep learning model tailored to specific requirements. Access to video data and knowledge about deep learning models makes monitoring of trout and salmon dynamic and hands‐on, as the collected data can be used to train and further improve deep learning models. Hopefully, this setup will encourage fisheries managers to conduct more monitoring as the equipment is relatively cheap compared with customized solutions for fish monitoring. To make effective use of the data, natural markings of the camera‐captured fish can be used for individual identification. While the automated process greatly reduces the need for human intervention in video surveillance and speeds up the initial sorting and detection of fish, the manual identification of individual fish based on natural markings still requires human effort and involvement. Individual encounter data hold many potential applications, such as capture–recapture and relative abundance models, and for evaluating fish passages in streams with hydropower by spatial recaptures, that is, the same individual identified at different locations. There is much to gain by using this technique as camera captures are the better option for the fish's welfare and are less time‐consuming compared with physical captures and tagging.

## Graphical abstract

This study outlines a method for using surveillance cameras and an algorithm that calls a deep learning model to generate video segments featuring salmon and trout in small streams. This automated process greatly reduces the need for human intervention in video surveillance. A comprehensive guide is provided on setting up and configuring surveillance equipment, along with instructions on training a deep learning model tailored to specific requirements. There is much to gain by using this technique as camera captures are the better option for the fish's welfare and are less time‐consuming compared with physical captures and tagging.

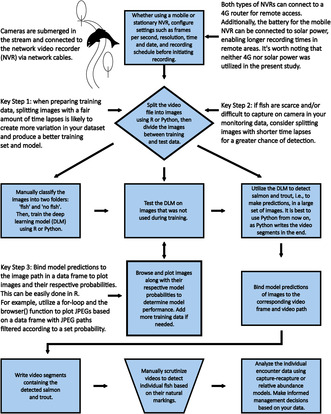

## 1 INTRODUCTION

Recent advances in the accessibility and relative ease of use of surveillance technology and deep learning models are shifting how ecological monitoring is conducted (Aguzzi et al., 2020; Saleh et al., 2020, 2023). Surveillance cameras can produce copious amounts of video and image data, and deep learning models can analyse the data with speed and accuracy (Hentati‐Sundberg et al., 2023).

A key benefit of this type of data is its cost‐effectiveness and scalability. Therefore, data can be collected from small systems that were previously neglected in ecological monitoring. This is particularly important for brown trout (*Salmo trutta*, hereafter referred to as trout) and Atlantic salmon (*Salmo salar*, hereafter referred to as salmon), two species that utilize streams and rivers as spawning and nursing grounds (Klemetsen et al., 2003).

For instance, in Sweden, where the present study took place, there are 111 defined catchment areas that are open to the sea along the Swedish coastline. The definition of a catchment area by SMHI (Swedish Meteorological and Hydrological Institute) is having an area of >200 km^2^. The Swedish Electrofishing RegiSter has data from 109 of these, where juvenile trout has been found in 103 and juvenile salmon in 61 (Sers, 2013, data extracted July 2022). The median size of the defined catchment areas where juvenile trout has been found is 588 km^2^, and for salmon, it is 1229 km^2^. Additionally, catchment areas of undefined size but less than 200 km^2^ are located on islands in the archipelago or wedged between the mouths of larger catchments in coastal areas. Out of 1902 water courses sampled in these small catchments, juvenile trout has been found in 1337 and juvenile salmon in 106 (Sers, 2013, data extracted July 2022). However, water courses were not sampled randomly, so the proportions between sampled water courses and the occurrence of trout and salmon may be misleading. In contrast, monitoring of adult trout and salmon focuses mainly on the greatest fluvial systems, often in conjunction with hydropower, or in systems where there is a public interest such as recreational angling (Hagelin et al., 2021; Shephard et al., 2019). Hence, in proportion of occurrence adult salmon is better monitored than adult trout. Therefore, there is a need to increase the coverage of ecological monitoring in small fluvial systems, and especially for trout.

Animal density and movements are fundamental estimates in applied ecology (Royle et al., 2013, Chapter 1). The methods used to estimate these parameters rely on data where individuals have been observed multiple times. If individuals can be unambiguously identified from frames in a video, this information can serve as capture histories in capture–recapture models to estimate population size (Karanth, 1995; Karlsson & Kari, 2020). Furthermore, if an individual is observed at different locations, this provides valuable information about its movements (Efford, 2004).

Since both trout and salmon are significantly impacted by the exploitation of fluvial systems and are threatened or extinct in many areas (Johnsen et al., 2011; Junge et al., 2014), it is important to continuously monitor these populations. This monitoring is important not only to establish a population baseline but also to assess whether measures taken, such as habitat restoration, improved connectivity or changes in fishing regulations, have had an effect.

The aim of this study was to describe how to set up, configure, and use Power over Ethernet (PoE) Internet Protocol (IP) cameras for monitoring trout and salmon in small streams and to analyse the videos using a deep learning model. Videos from the cameras were recorded on either stationary or mobile battery‐driven network video recorders (NVR), depending on whether the sites had access to the local electric grid. Furthermore, I describe how to analyse the videos in Python and, to some extent, in R (R Core Team, 2023). Currently, R does not have as powerful video processing capabilities as Python. In both R and Python, I trained deep learning models to detect fish and, finally in Python, wrote an algorithm to automatically generate short video segments of the detected fish (a link to data and the algorithm is provided). The purpose of the algorithm was to identify the occurrence of trout and salmon in large datasets and further, to generate video segments that produce sequences where the fish are clearly visible with enough sharpness and focus for manual individual identification if captured on camera again. The resulting predictions from the deep learning model are presented to provide a general idea of its performance and possible applications.

## 2 METHODS

### 2.1 Setting up the network video recorder, mobile NVR and Internet Protocol cameras

The IP cameras were of the Linovision 4 K PoE IP underwater camera anti‐corrosion type, equipped with either 30‐ or 50‐m cables. Cameras were mounted on iron fundaments to secure them in the stream; network cables were weighed down to the river bottom by chains to prevent drifting in the stream and avoid snagging debris.

The cameras were connected to either a battery‐powered mobile NVR or a stationary NVR, depending on if the sites of recording had access to the local power grid. Recordings were made at 3840 × 2160 resolution and 20 frames per second. A detailed description of how to set up the NVRs and cameras is found in the Appendix A.

### 2.2 Recording

Videos of salmon and trout were recorded at three different locations. Skeboån is a small stream where sea trout migrate to spawn in late autumn. (Sea trout is a migratory form of brown trout that migrate from freshwater rivers and streams to the sea, where they spend part of their life before returning to freshwater to spawn.) Here, an association of recreational anglers has restored spawning habitat and stocked trout. In Skeboån, I recorded on a restored spawning ground in Häverödal during the spawning season in late October 2023 (Latitude: 60.025197, Longitude: 18.603479).

The Dalälven River has a governmentally funded hatchery with salmon and sea trout. This river is regulated by hydropower, so densities of spawning fish are very high below the lowermost migration barrier at Älvkarleby, where I recorded spawning fish in late October 2023 (60.563282, 17.435323).

Mörrumsån is a small river famous for its recreational angling of salmon and sea trout. Here, fish begin their upstream migration in early summer, while they are still silvery and have not yet developed their spawning coloration. I recorded near ‘Laxens hus’ in Mörrum town during the second half of July 2023 (56.192046, 14.748952).

### 2.3 Video processing and training of the deep learning model in R and Python

Both R and Python were used separately to generate images from video frames. However, I recommend using Python due to its faster processing speed and simpler syntax. In R, videos were processed using the ‘av’ package (Ooms, 2022), with the ‘av_video_images’ function was used to extract one frame for every 20 s of video, effectively thinning the videos. In Python, the ‘ffmpeg’ software and module (Tomar, 2006) were employed to generate images at 20‐s intervals from the video using the ‘input’ and ‘output’ functions. Both R and Python functions produce images at the specified interval, but there may be differences in the exact start or end frames utilized, resulting in potentially distinct images. All recordings used to train and validate the model were captured at 20 frames per second. Approximately 3 h of continuous video material from one camera in Skeboån was used. In Mörrumsån, 5 + 5 + 16 + 16 h of continuous videos from two cameras (5 + 37 h) for 3 days were used. In Dalälven, 4 h of continuous video from one camera was used to train the model.

Each image was reviewed by me to determine the presence of a fish (or part of a fish) and then classified into one of two folders: ‘fish’ or ‘no fish’. Images from each location and camera were compiled into these folders. For some images, it was challenging to discern whether a fish was present; they included only blurry fragments. In other images, the fish was sharp and in focus. However, I chose not to train the model exclusively on ‘ideal images’ but on all images produced by splitting the training videos. This approach ensures the inclusion of real‐world complexity in deep learning models for effective real‐world applications (Saleh et al., 2020). With the 20‐s thinning of the videos, this resulted in 8960 images, of which 7772 did not include a fish, and 1188 included a fish or parts of a fish. The classification of the training data revealed images only of trout and salmon; no other fish species were identified.

The model was trained on Windows 10, both in R using a Python environment with the packages Reticulate (Ushey et al., 2023), Keras (Allaire & Chollet, 2023) and TensorFlow (Allaire & Tang, 2023), and in Python using a Conda TensorFlow environment. The same model architecture was employed in both R and Python; however, the syntax differed between the two languages. The training and validation images were pre‐processed and loaded using the ImageDataGenerator. A base model with the Xception architecture, utilizing pre‐trained weights from ImageNet (Chollet, 2017), was employed. The layers of the model were frozen to prevent further training. A new sequential model was constructed on top of the base model, incorporating a global average pooling layer, a dense layer with ReLu activation, a dropout layer and a final dense layer with softmax activation for classification.

The model was compiled using categorical cross‐entropy loss, the Adam optimizer with a specified learning rate, and accuracy as the evaluation metric. The batch size and epochs were set to 32 and 3, respectively, and the model was trained using the specified training and validation generators. Eight different models were fitted, encompassing all unique combinations of the hyper‐parameters: learning rate (10^−2^ or 10^−3^), dropout rate (0.2 or 0.3) and the number of neurons in the dense layer (256 or 1024). Hyper‐parameters were based on default values and experimental refinement for the specific task and data at hand. The model with the highest validation accuracy after three epochs achieved approximately 93% accuracy (learning rate: 10^−3^, dropout rate: 0.2 and neurons: 256) and was selected for testing the data. There was, however, only a 1–2% difference between the worst and best model, and there is a level of stochasticity in the model training. Therefore, the exact hyper‐parameter values within this range may not have been important in this particular case. You can find a link to the code for setting up the model in R and Python under the data availability section.

### 2.4 Testing the model

To test the model, I utilized videos from Mörrumsån, covering a total of 6 days from 22 to 27 July, with recording sessions between 05:00 and 21:00 each day. These hours ensured there was sufficient light to observe the fish clearly. Similar to the process used in creating the training and validation data, the videos were thinned into one image every 20 s. The model was then employed to predict each image and provide a probability of whether the image included a fish or not.

The predictions were summarized in a table where each row represented a unique probability (e.g., 0, 3, 25 and 100), and the columns represented specific dates. The frequency of images per unique probability per date was matched to the corresponding row. All images with a probability of ≥3% were scrutinized by me to determine whether they contained a fish or not. Images with a probability of <3% were not scrutinized. Due to the large number of images with a <3% probability and the indication from images with ≥3% probability that, if the <3% images contained a fish, the image would be of very poor quality and the information would be largely meaningless. A subset of the images was selected and plotted along with their respective probabilities to provide a general idea of how well the model performed.

### 2.5 An algorithm to write video segments of the detected fish

As described above, the model predicted the images, and their respective probabilities were stored in a column of a data frame, along with a column containing the path to each image. Probabilities and image paths were arranged in chronological order. Using the ffmpeg function ‘probe’, the number of frames per video and the frame rate of the videos (20 frames per second) was calculated. Knowing this, and having all the probabilities and image paths sorted chronologically with 20 s between images, it was possible to determine the corresponding frame in each video from which the image prediction was made.

For each image (row) in the data frame, the frame number, the path to the video and the total frame number of the video were added. Furthermore, a sequence from 1 to the number of images in each video file was included as a column in the data frame, making it possible to determine whether two adjacent images both contained a fish (a true or false statement; if the difference between two images is >1, they are not adjacent).

The data frame was then filtered based on a set probability, for example, ≥50%. The resulting data frame, consisting of video frames with a probability of ≥50% to contain a fish, was split by video file, creating a separate data frame for each video file. Within each data frame, adjacent video frames containing a fish were assigned to a ‘group’ column. The split data frames were concatenated, and the resulting data frame was split a second time, now by video and group. Each data frame now represents video frames from the same fish observation, where the number of rows depends on how long the fish has been in front of the camera.

For each data frame, the minimum and maximum frame numbers were selected and used as start and end frames. A buffer of 20 s was set for start and end frames, achieved by subtracting and adding 400 frames to the start and end frames, respectively. The start frame was set to a minimum of 1, and the end frame was set to a maximum of the video's total frames, to avoid going out of bounds. The video path, start frame and end frame were then used by ffmpeg when writing the video files. To test this algorithm, I applied it to the videos from 22 to 27 July in Mörrumsån, encompassing 16 h of video per day. The algorithm performed as expected on all tested dates; however, in the results, enclosed data and algorithm, I present the analysis from 26 July, as this day had the most camera captures of fish.

## 3 RESULTS

**FIGURE 1:**
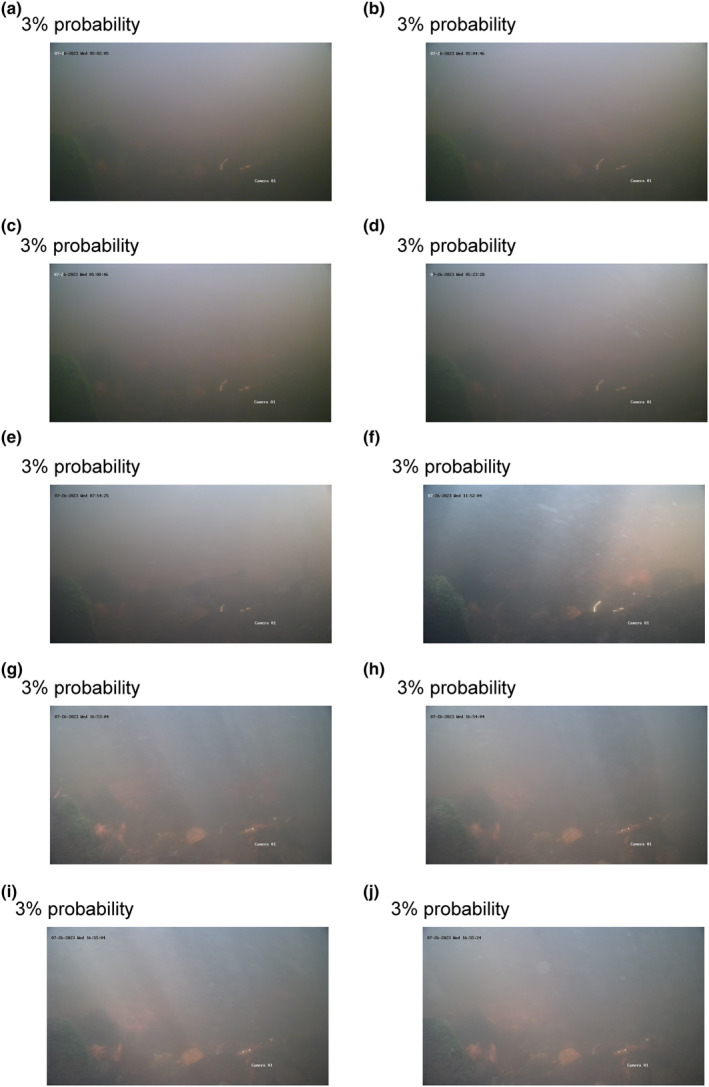
Ten images (a–j) from 26 July, each having a 3% probability that the image contains a fish. In image (e), there is a weak silhouette of a sea trout or a salmon; in the remaining images, there are no fish.

**FIGURE 2:**
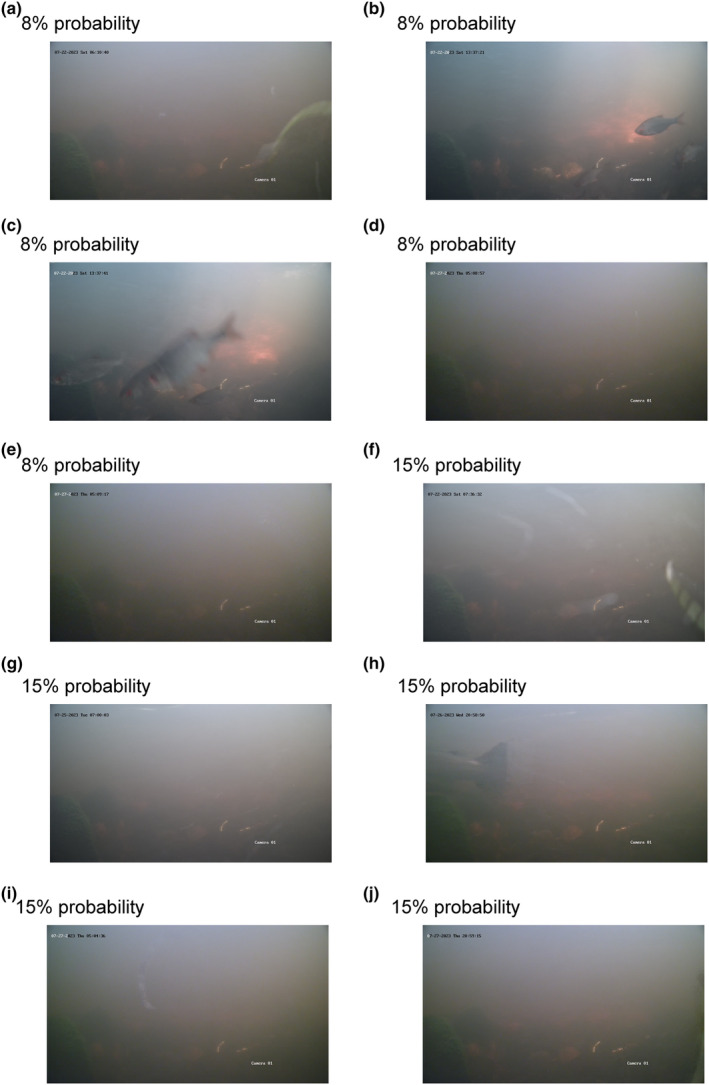
Five images with an 8% probability that they contain a fish (a–e). In panels (b, c), there is a group of cyprinids that the model cannot detect as fish. In images (f–j), each have a 15% probability of containing a fish. In image (h), there is a blurry tail of a salmonid.

**FIGURE 3:**
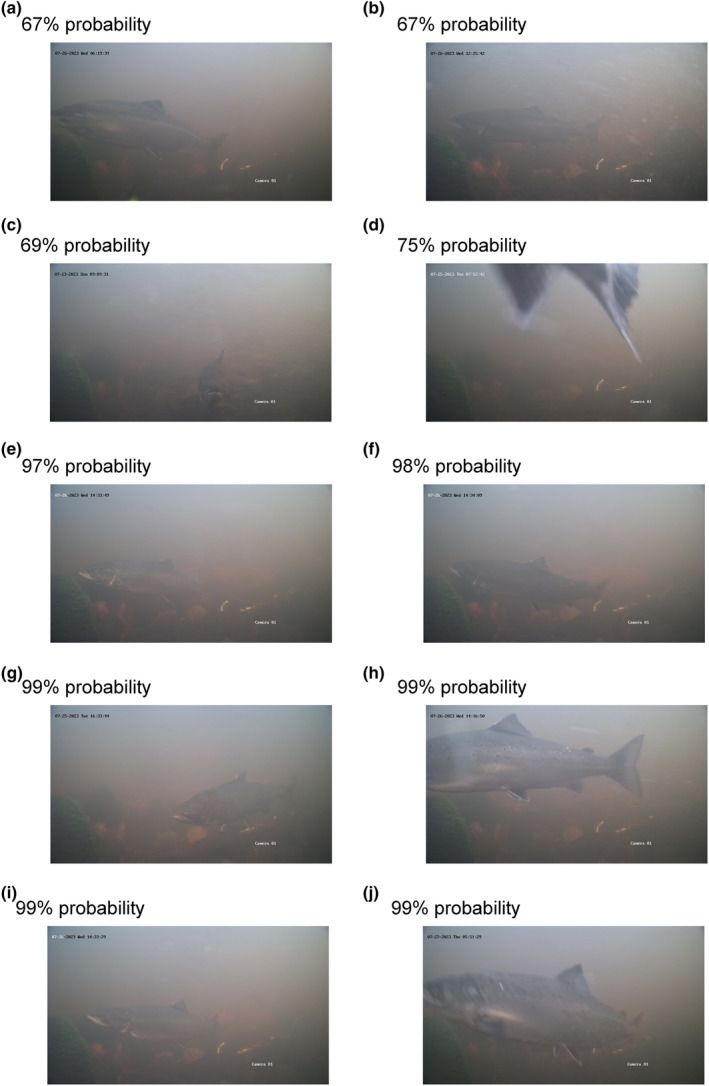
Ten images (a–j) with a 67% to 99% probability of containing a fish, and each image clearly contains a fish or parts of a fish.

The model was used to predict a probability for each of the 17,296 images between the dates 22 and 27 July in Mörrumsån. Here, 0 indicates no probability, and 100 indicates full probability that the image contains a fish or part of a fish. The results showed an increasing proportion of correctly classified images with increasing probability that the image contained a fish (Table 1). As the probability decreased, the proportion of images including a fish decreased, and the clarity of the fish on the images that did contain a fish gradually worsened with lower probabilities (Figures 1, 2, 3).

**TABLE 1:** In total, 17,296 images, or 96 h of video (16 h * 6 days), were predicted and given a probability that the image contained a fish.

Prob. is fish	July 22	July 23	July 24	July 25	July 26	July 27	Is fish?
0	2215	2451	2360	2290	2443	2211	Not checked
1	474	375	438	509	361	497	Not checked
2	112	28	46	58	46	71	Not checked
3	50	17	23	13	10	25	5/138
4	12	8	12	2	6	22	No fish
5	1	3	1	1	2	16	2/24
6	6	–	1	1	1	9	No fish
7	2	–	–	2	1	10	No fish
8	3	–	–	–	–	2	2/5
9	–	–	–	–	1	3	1/4
10	1	–	–	1	–	1	No fish
11	–	2	–	1	–	1	No fish
12	–	–	–	–	–	1	No fish
13	–	–	–	–	–	1	No fish
14	–	–	–	–	–	3	No fish
15	1	–	–	1	1	2	1/5
16	–	–	1	–	–	1	No fish
17	2	–	–	–	1	–	1/3
27	–	–	–	–	1	–	Fish
28	–	–	1	–	–	–	No fish
34	–	–	–	–	–	1	No fish
35	–	–	–	–	1	–	Fish
39	–	–	–	–	1	–	Fish
64	–	–	–	–	1	–	Fish
67	–	–	–	–	2	–	Fish
69	–	1	–	–	–	–	Fish
75	–	–	–	1	–	–	Fish
97	–	–	–	–	1	–	Fish
98	–	–	–	–	1	–	Fish
99	–	–	–	1	2	1	Fish
100	1	–	–	–	6	–	Fish

*Note*: The leftmost column represents these probabilities, while the columns with dates show the frequency of images in each probability category (the probabilities were multiplied by 100 and rounded to the nearest whole number). The rightmost column, labelled ‘is fish?’, indicates whether the images in each category actually contained a fish. If a proportion is stated, it represents the number of images with a fish relative to the total number of images. The probability categories 0, 1 and 2 were not checked as it would be too laborious. To get an idea of what type of images, the model can and cannot handle, images from the coloured cell and rows are shown in the figures below. For 3% probability during 26 July, see Figure 1, for 8 and 15% probability, see Figure 2, and for 67 to 99% probability, see Figure 3.

The grey shaded cell represent the images shown in Figure 1, the blue shaded cells represent images shown in Figure 2, and orange shaded cells represent the images shown in Figure 3.

**FIGURE 4:**
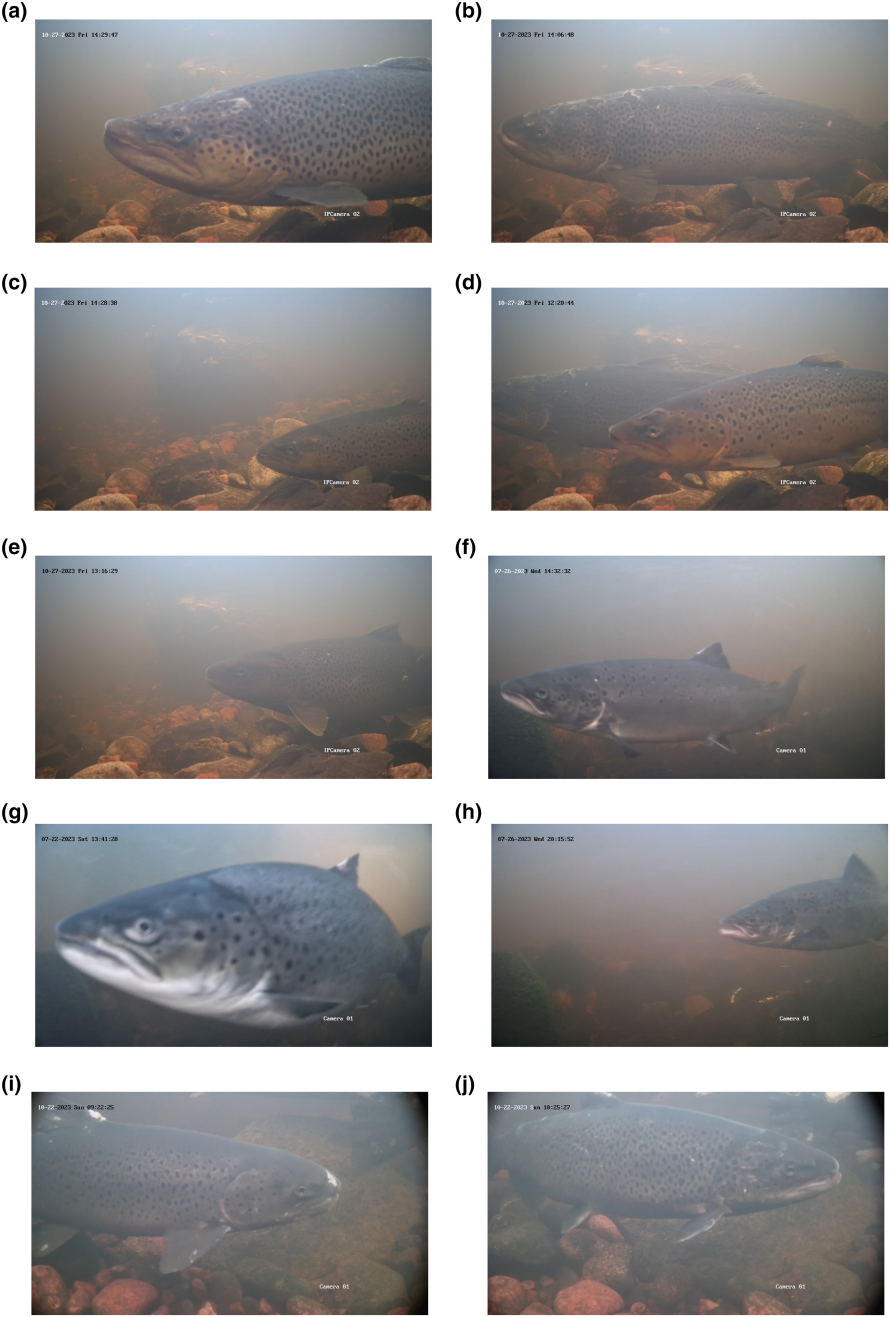
Images where the fish's head is sharp and in focus: (a–e) sea trout in Skeboån; (f) salmon; (g) sea trout; (h) salmon, all in Mörrumsån. Panels (i, j) show either salmon, sea trout or hybrids of the two (I cannot determine which) in Dalälven. Each image show an individual fish.

Ten images, where the fish was sharp and in focus, were selected to illustrate the type of images that could be used for individual identification (Figure 4).

From the video files of 26 July recorded in Mörrumsån, the algorithm produced six video files of salmon, three of which are shown here (Videos 1, 2, 3).

**VIDEO 1:** Salmon swimming downstream and stopping in front of the camera. Video segments are created between 400 frames (20 s) before and after the frame in which the fish is detected by the deep learning model.

**VIDEO 2:** This specific salmon was recorded four times on July 26th, it is the same individual as in Video 3. Video segments are created between 400 frames (20 s) before and after the frame in which the fish is detected by the deep learning model.

**VIDEO 3:** This specific salmon was recorded four times on July 26th, it is the same individual as in Video 2. Video segments are created between 400 frames (20 s) before and after the frame in which the fish is detected by the deep learning model.

## 4 DISCUSSION

This study demonstrates the assembly of NVR and IP cameras for recording trout and salmon in small streams. Additionally, it illustrates how to detect trout and salmon in the video by training a deep learning model for image classification and, finally, how to produce video segments of the fish. The key benefit of this monitoring approach is that physical captures and tagging of the fish are not necessary, thus avoiding any impact on fish welfare. Moreover, the method is relatively straightforward, and NVRs and IP cameras offer a cost‐efficient alternative to customized solutions for fish monitoring provided by commercial companies, especially when scaling up the monitoring efforts. Upscaling is facilitated as the NVRs support up to eight IP cameras, and the number of cameras connected to the NVR can be increased using a PoE switch. Because the cameras are small, relatively inexpensive and easy to handle, they can be deployed in high numbers directly in the stream in areas where the fish are believed to pass. This reduces the need to make changes in the riverbed, preserving the fish habitat. Another benefit of this method is that those conducting the monitoring have complete access to all video data, the algorithm and the deep learning model. These resources are typically not provided by commercial companies that offer proprietary software and licenses. Data availability allows the user to further train the model based on their needs, leading to improved monitoring and deep learning models (Saleh et al., 2023). Hence, the methods described here align with modern perspectives on animal welfare, habitat protection and the transparency of open science.

Recent advances in camera technology have accelerated its application in applied ecology (Royle et al., 2018). The data presented in this study can be utilized in various ways, such as in capture–recapture and relative abundance models. Both types of models rely on individual identification. The main difference between the two is that the former relies on recaptures to estimate a detection probability that is related to the number of captured individuals (Kéry & Royle, 2021, introduction chapter). Hence, recaptures transform a relative abundance into an unbiased population size, essentially providing a detection correction to the relative number. In studies of feline mammal populations, camera traps are commonly employed to gather data, relying on the identification of individuals through their natural markings (Karanth, 1995). This method of individual identification has also found its way into fisheries science, where individual pike (*Esox lucius*) can be distinguished based on their conspicuous markings (Karlsson & Kari, 2020). Consequently, documenting daily camera captures of trout and salmon through videos provides the necessary individual encounter data for capture–recapture and relative abundance models. Providing estimates that are fundamental for the management of these populations.

A further application of this type of setup is the evaluation of fish passages. Dams for hydropower and water storage block the migration of anadromous and potamodromous fish such as trout and salmon, that is, fish that migrate from freshwater rivers to the ocean or large lakes and back to spawn (Klemetsen et al., 2003). One approach to guiding fish past these dams is by creating fish passages, typically small side streams that circumvent the dam and connect to the river upstream. However, enticing fish to use these passages is challenging as they often prefer to remain in the mainstream where water flow is highest. From a fish's perspective, the mainstream is likely the most direct route to their spawning grounds, making them reluctant to enter smaller side streams, such as fish passages (Silva et al., 2018). Consequently, the construction of fish passages does not guarantee their utilization by fish, necessitating continuous evaluation and adaptation of these structures (Silva et al., 2018). The presented setup could be employed for fish passage evaluation, monitoring the entry and progression of fish through the passage, and measuring their passage time—three essential metrics for evaluating fish passages, as highlighted by Silva et al. (2018). This evaluation could be conducted by placing cameras downstream of a fish passage to observe whether individuals captured on camera downstream entered the fish passage upstream. Additionally, cameras placed at the ends and along the fish passage could be used to estimate passage time. By using natural markings and cameras to identify the fish, spatial recaptures can be used to track its movements. This is harmless to the fish and aligns with modern views on fish welfare (Kristiansen & Bracke, 2020). This approach stands in contrast to conventional methods for fish passage evaluation, such as telemetry, which involves capture, sedation and surgery to implant transmitters in fish for monitoring their movements (Cooke & Wagner, 2004; Silva et al., 2018).

### 4.1 Optimizing system usage: Practical suggestions and limitations

The presented deep learning model is trained to detect salmon and trout. With the model's predictions, it is possible to filter out images where the fish is clearly visible. These images are associated with video frames and the video itself, which are then used to generate video segments. These segments are intended to facilitate the unambiguous identification of individual fish based on their natural markings through manual scrutiny. For this process to be feasible, the number of detected fish cannot be too high, as it would result in extensive manual labour. For reference, Karanth (1995) utilized 31 camera captures of tigers (*Panthera tigris*) in Nagarahole National Park, India, to unambiguously identify individuals. Similarly, Karlsson and Kari (2020) had 66 camera captures of pike (*Esox lucius*) from a small Swedish lake for individual identification. In the far end of the spectrum, in a catalogue of 850 photo‐identified right whales (*Eubalaena* sp.), a single matching attempt may take up to 3 h, which was considered about the maximum practically for that researcher (Hammond et al., 1990). Although 3 h for a single matching attempt likely far exceeds what can be considered feasible in many research projects, quantities up to around 100 captures may represent a more typically manageable volume for manual identification until automated processes are implemented. Based on those numbers, this method renders it feasible to survey, for example, the total population of a small stream or the spawning population within a geographically defined spawning area in a somewhat larger fluvial system.

It is important to clarify that capture–recapture and relative abundance models do not depend on capturing all individuals (Kéry & Royle, 2021, introductory chapter). This means that, for example, the inability to capture individuals during night‐time does not inherently pose a problem. However, factors such as day length and the operational time of cameras may need to be considered in the model. Similarly, environmental changes such as water flow or temperature can alter the fish's movement in relation to the camera, thereby affecting capture probability, which may necessitate adjustments in the model. Additionally, changes in camera location, orientation and the number of cameras deployed will also impact capture probability and may require adjustments in the model.

Furthermore, achieving unambiguous identification of captured fish will not always be possible, even if the fish is captured on camera. This could be due to various reasons, such as the fish quickly sprinting past the camera, being too distant from it, or presenting the opposite flank for identification than what is required. Hence, in these cases, and many others, where the fish cannot be unequivocally identified, I suggest omitting these observations and considering the fish as not detected. Some of this variability may be addressed by incorporating covariates in the model, such as water flow, which can affect the duration and proximity of fish to the camera. Consequently, changes in the detection probability can be explicitly accounted for during capture–recapture and relative abundance modelling.

Changes to the settings of the NVR and algorithms can potentially influence capture probability. For example, video compression techniques are known to exploit temporal redundancy by encoding frames differentially or by referencing neighbouring frames, indicating a potential dependence of each frame's information on data from adjacent frames. Consequently, it is theoretically plausible that recording at a higher frame rate per second could enhance the likelihood of acquiring high‐quality images suitable for individual identification. However, the precise impact of this effect, along with other factors related to the NVR's configuration such as image resolution and exposure settings, remains unclear. It is necessary to assess whether these factors are significant and they should be considered given the current conditions, especially if settings are changed during the study. This is because the quality of images and the prevailing circumstances when images are taken are key to the successful identification of animals based on natural markings (Gunnlaugsson & Sigurjonsson, 1990; Würsig & Jefferson, 1990). Therefore, it is important to have these factors in mind when configuring the NVR and analysing the data.

Furthermore, the time lapse between images when splitting the video files of the monitoring data is crucial as it directly impacts detection probability. In the provided algorithm, a 20‐s interval was utilized, which can be easily adjusted if necessary. A shorter time lapse results in more detections, whereas a longer time lapse leads to fewer detections. This aspect is also influenced by the duration fish spend in front of the camera. For instance, during recordings at spawning sites, I observed fish that remained stationary for several minutes, only to briefly leave and then return to the same location. Conversely, during migration in the lower reaches of a river system, fish may only briefly pause in front of the camera before moving on.

Reducing the number of detections may be advantageous if detections are overly abundant, as this alleviates workload, provided there is sufficient data. However, it is crucial that the thinning process remains consistent throughout the study, or that any changes in the time lapse between images should be accounted for as covariates in the capture–recapture or relative abundance model. Therefore, when thinning the video files, prevailing conditions must be considered.

However, the same strict adherence to thinning does not apply when splitting the video files for training data. Here, the primary goal is to avoid overly similar images to increase dataset variation. A 20‐s time lapse allows sufficient time for objects such as bubbles, debris and plants on rocks, as well as the fish itself, to change positions in the image. This variation reduces the risk of overfitting and artificially inflating the model's validation accuracy. Overfitting occurs when the model performs well on validation data, which contains similar images, but fails to generalize to unseen data (Borowiec et al., 2022; Shorten & Khoshgoftaar, 2019; Ying, 2019). Moreover, incorporating data from different recording locations, such as Skeboån, Dalälven and Mörrumsån in the present study, as well as from different cameras within the same stream, is vital in creating dataset variation.

Another parameter that affects detection probability and can be adjusted in the algorithm is the probability filter when writing the video segments. In the current study, this was set to ≥50%. A higher probability will result in a greater proportion of videos of good quality but will inevitably overlook detections that were of good quality by chance, as the image the deep learning model based its predictions on may not fully represent the entire video sequence. Therefore, similar to the thinning of video files, prevailing conditions must be considered when using this filter. It must be considered whether changes to this setting should be incorporated into the capture–recapture or relative abundance model as they will introduce variation in detection probability.

### 4.2 Suggested statistical analyses and exemptions from statistical analyses

A significant challenge in estimating the populations of migrating animals arises from the fact that these populations are not closed. There is a constant flow of immigration and emigration within the study area, thus violating one of the core assumptions of capture–recapture models, which assumes equal capture probabilities for all individuals. One model that can be used to monitor the population size of migrating salmon is a three‐part hierarchical model: a conditional multinomial, followed by a binomial model, and finally a Poisson model. In short, this model makes use of two capture locations, one downstream and one upstream, and assumes that individuals mix in between so that the capture probability between the two locations is independent. This model is described in various versions in Schwarz and Dempson (1994), Mäntyniemi and Romakkaniemi (2002), and for an application not relating to migrating fish in Chapter 7 of Kéry and Royle (2016).

Another approach is to utilize the spawning period when salmon and trout are stationary, conducting the capture–recapture study on spawning grounds during the brief period when the population is closed. While this allows the model assumptions to be met, it does not enable us to estimate the entire population of the fluvial system. This approach was employed in a study of Atlantic sturgeon (*Acipenser oxyrinchus oxyrinchus*) in the Hudson River, as well as in studies of migrating birds like the willow warbler (*Phylloscopus trochilus*), excluding time periods when fish and bird populations were highly transient (Kazyak et al., 2020; Chapter 3 in Kéry & Royle, 2021).

A final suggestion is to exempt statistical analyses and simply count the number of observed unique individuals during the study period. This may prove beneficial in small streams where sea trout migrate to spawn in the autumn. This is because, unlike large fluvial systems, spawning migration and spawning in small streams typically occur within a short timeframe (Klemetsen et al., 2003). For example, members of the Recreational Anglers Association in Skeboån reported that migration and spawning typically conclude within a month. Moreover, most individuals of the population can likely be observed if the stream is small enough and if multiple cameras are used. This is crucial as much of the monitoring is conducted by fisheries associations that may lack the resources to perform advanced statistical analyses and adhere to strict requirements on data collection.

### 4.3 Future directions

Throughout all stages of the analysis process, Python consistently outperformed R in terms of speed, rendering the use of R unnecessary for this type of analysis. Given that R cannot generate the video segments, which serve as the final output of this workflow, it may be worthwhile to entirely bypass its usage. Additionally, I encountered difficulties when attempting to load the deep learning model trained in R into Python, necessitating the retraining of the model in Python. While this posed a minor inconvenience, as the R code for training the model can be easily translated into Python, I initially utilized R out of convenience and habit to generate tables and figures based on model predictions for evaluating model performance. Therefore, while there is some value in having the deep learning model in R, particularly if R and RStudio are the preferred programming language and integrated development environment, it is not strictly necessary.

As additional features, the battery powering the mobile NVR can be connected to solar power, which can simultaneously charge the battery while it is in use. This addition may prove cost‐effective in the long run, as it reduces the need to change batteries in remote areas. Additionally, both types of NVRs can connect to a 4G router, allowing remote access. This remote access capability can serve as a cost‐effective method to monitor that data collection is progressing as desired, without the need to be physically present. However, neither a solar‐powered setup nor 4G access were considered in the present study due to budget constraints and short‐term needs. Nevertheless, their potential benefits for future and extended monitoring are compelling.

The success of future monitoring depends on the strategic placement of cameras, as the image quality and number of camera captures varied notably between locations. In Mörrumsån, for instance, the cameras were positioned at the end of a 200 m stretch of rapids and white water, resulting in images with a white tone due to air bubbles that limited visibility. In contrast, in Skeboån, the camera was situated at the beginning of a small rapid without any white water that served as a spawning ground, yielding much clearer images of stationary fish. However, in Mörrumsån, the fish circulate below the rapids before starting to climb and are hence easily captured on camera compared with other areas in this river, introducing a trade‐off between image quality and quantity when placing cameras in this area. Thus, the placement of cameras emerges as an important factor for the success of this monitoring approach.

To further utilize the data, the resulting video segments from the model and algorithm presented here could be split into images. This is to train a second model to select images containing at least the head and upper body of the fish in good resolution, and showing either the left or right flank of the fish. Subsequently, a third model could use these selected images and be trained for species classification and, finally, to classify individuals. However, training the suggested models would require the collection of more data and raise more funding than what was obtained in the present study.

**FIGURE 5:**
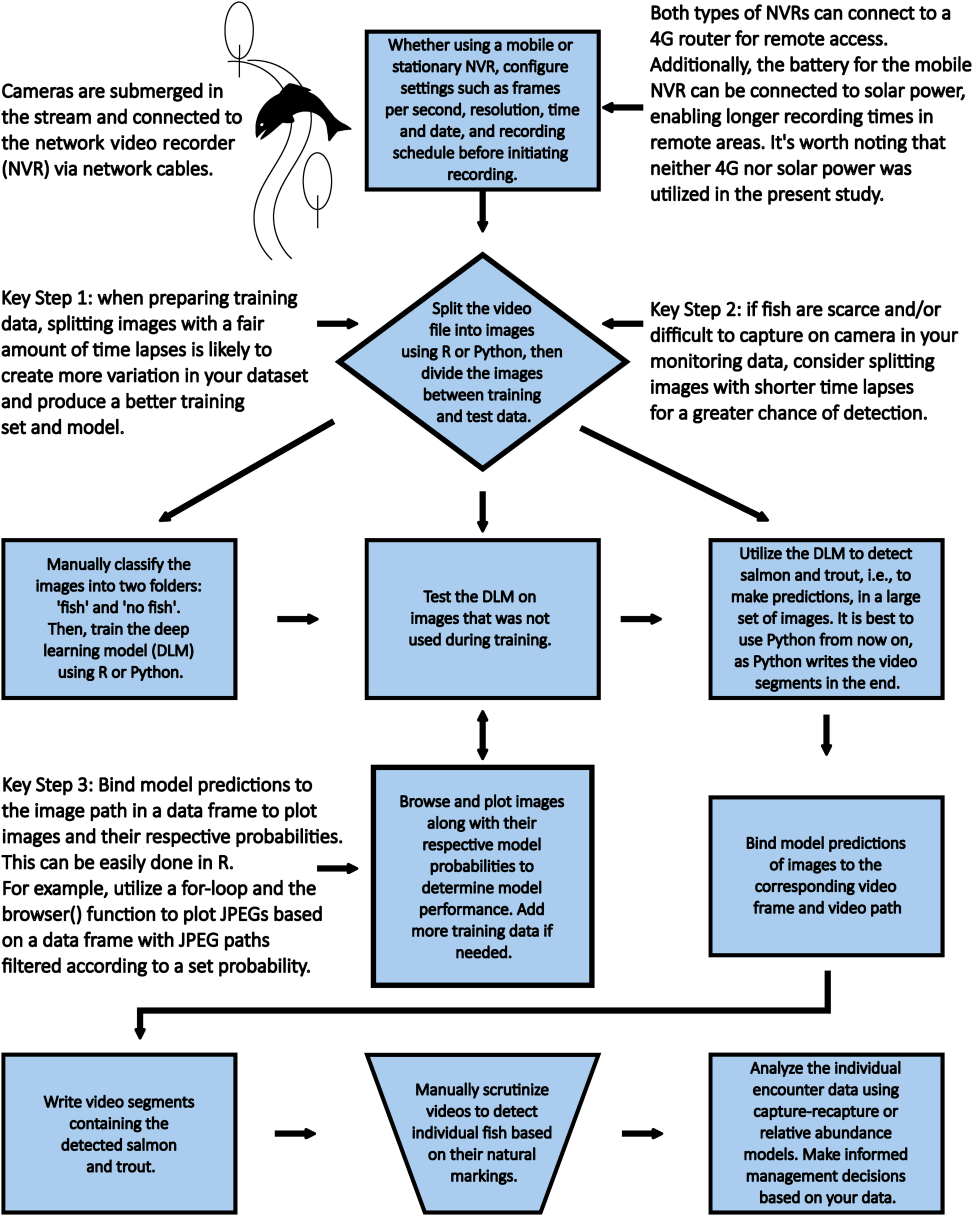
Suggested data collection and analysis workflow based on insights drawn from this study.

Based on the lessons learned from this study, a suggested workflow for data collection and analysis is summarized in a flow chart (Figure 5).

## AUTHOR CONTRIBUTIONS

**Konrad Karlsson:** Conceptualization (lead); data curation (lead); formal analysis (lead); funding acquisition (lead); investigation (lead); methodology (lead); project administration (lead); resources (lead); software (lead); supervision (lead); validation (lead); visualization (lead); writing – original draft (lead); writing – review and editing (lead).

## FUNDING INFORMATION

The study was funded by an internal grant at the Swedish University of Agricultural Science; Utvecklingsprojekt för fortlöpande miljöanalys (FOMA), miljöanalysprogram sjöar och vattendrag. That is, a development project for continuous environmental analysis.

## CONFLICT OF INTEREST STATEMENT

The author declares no conflicts of interest. The conclusions and opinions presented here are those of the author and do not represent the official position of the Swedish University of Agricultural Science. Any use of trade, firm or product names is for descriptive purposes only and does not imply endorsement by the Swedish University of Agricultural Science.

### OPEN RESEARCH BADGES

This article has earned Open Data and Open Materials badges. Data and materials are available at 10.5061/dryad.v6wwpzh3g.

## Figures and Tables

**FIGURE A1:**
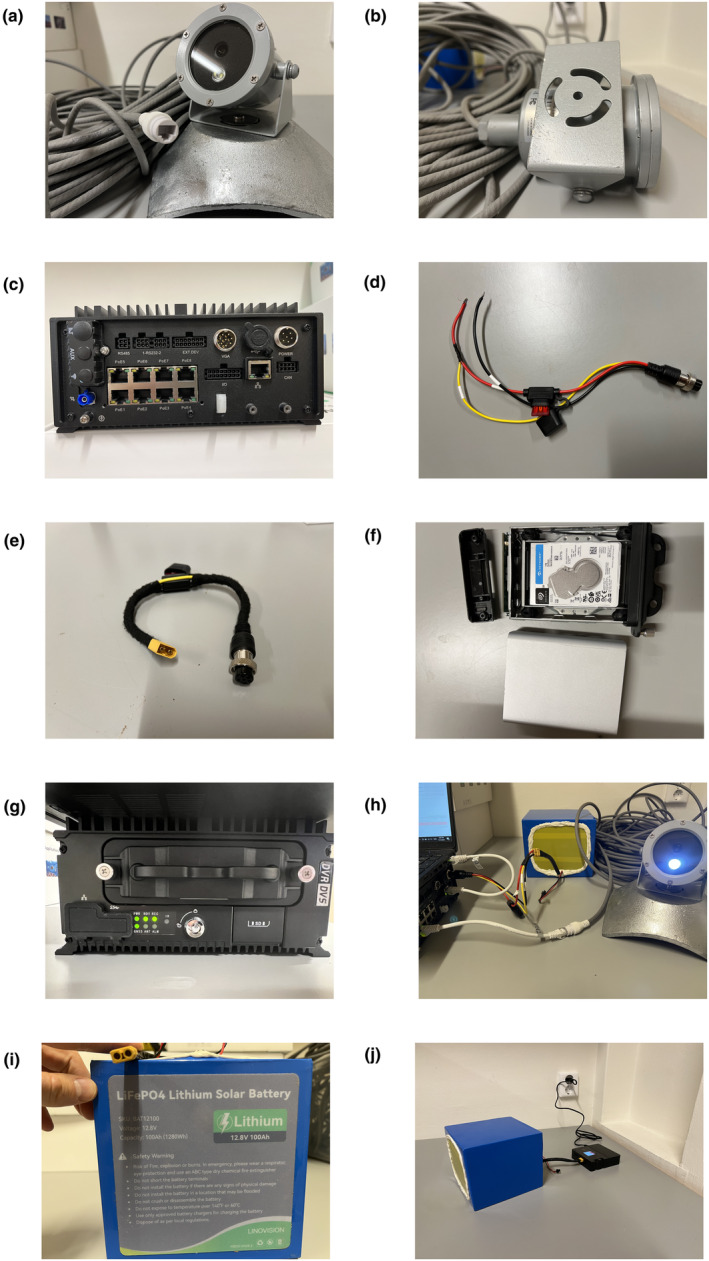
Panels (a, b) show the camera and its mounting on a lead fundament. Panel (c) displays the back of the mobile network video recorder (NVR) with eight connections for power over Ethernet (PoE), LAN and power. Panels (d, e) showcase the aviation power plug and the 10 A flat fuse. In (d), the yellow accessory wire has been connected to the positive red wire, while in (e), an XT60 connector has been soldered to the wire ends for easier connection to the battery. Panel (f) shows a 1 TB HDD mounted in a casing. In panel (g), the HDD casing is in the locked position, and the mobile NVR is powered and recording. Panel (h) essentially illustrates the entire setup, where the camera connects to the mobile NVR, the mobile NVR connects to the laptop via LAN, and the battery is connected to the mobile NVR. Panels (i, j) depict the battery and XT60 connector, with the battery charging using the ISDT® 608 AC smart charger.

**FIGURE A2:**
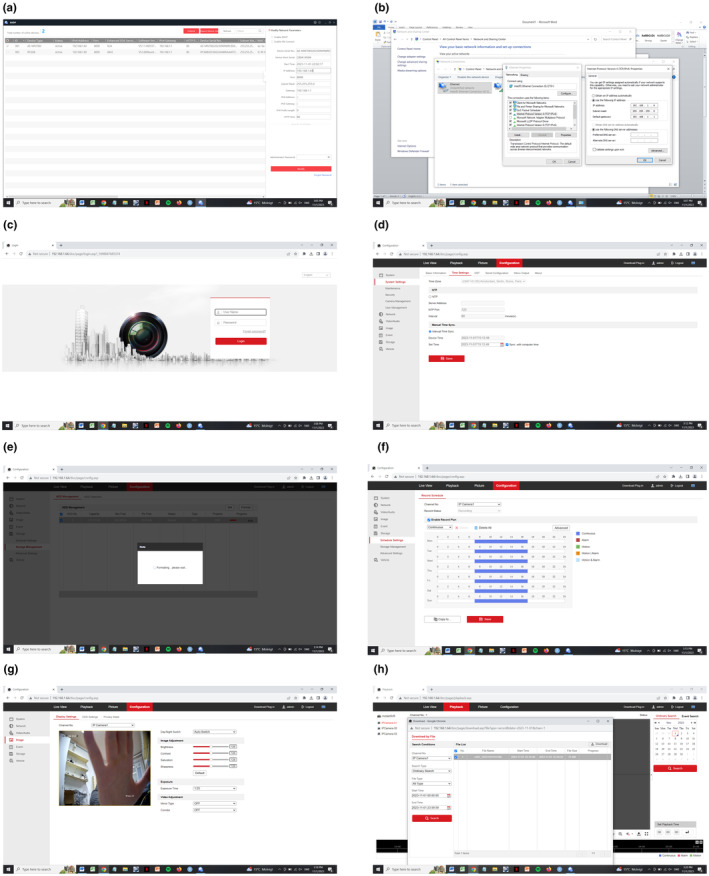
Panel (a) shows the SADP software with two active devices: a mobile network video recorder (NVR) and an Internet Protocol (IP) camera. These devices are activated by setting a password. Panel (b) demonstrates how to change and set the IP address, subnet mask and gateway on a laptop running Windows 10. Panel (c) illustrates the web browser login process. Panel (d) shows how to sync the mobile NVR's system time with the laptop. Panel (e) demonstrates how to format the HDD, so it can be used for recording. Panel (f) displays the time window for recording; in this case, the mobile NVR was set to record between 7 am and 5 pm every day. Panel (g) explains how to get a live view, while panel (h) shows how to download videos from the mobile NVR.

**FIGURE A3:**
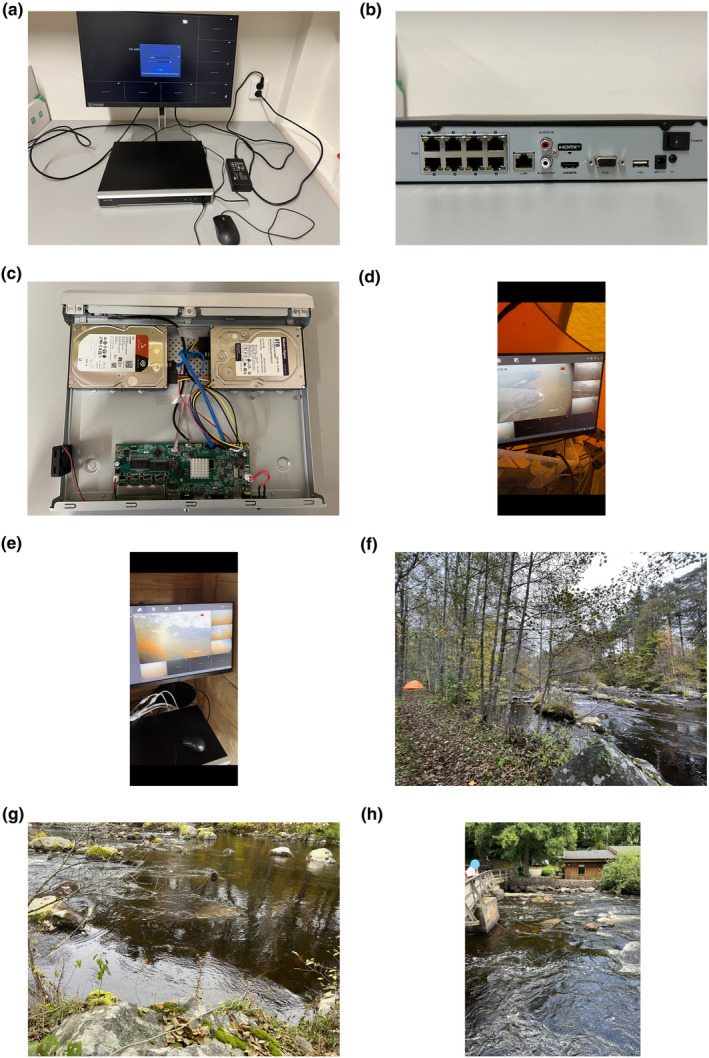
Panel (a) shows the network video recorder (NVR) connected to a monitor, power and a mouse. Panel (b) displays the back of the NVR with connections for power over Ethernet (PoE), LAN, HDMI and USB, among others. Panel (c) reveals the inside of the NVR mounted with two 8 TB HDDs. Panels (d, e) depict the NVR in use in a tent (Dalälven) and a shed (Mörrumsån), respectively. Panel (f) shows the tent raised close to a power source, with grey network cables extending from the tent down to the cameras in the river in panel (g). Panel (h) illustrates grey network cables hanging from a bridge in Mörrumsån, connecting to cameras in the river.

## Data Availability

Data and code are publicly available at the Dryad data repository DOI: 10.5061/dryad.v6wwpzh3g.

## APPENDIX A SETTING UP THE NETWORK VIDEO RECORDER, MOBILE NVR AND INTERNET PROTOCOL CAMERAS

The Internet Protocol (IP) cameras and network video recorders (NVR) used in this study adhere to the Open Network Video Interface Forum (ONVIF), an open standard for IP‐based security products, allowing products from different manufacturers to communicate with each other. This means that similar devices, as presented below, can be used instead, as long as they adhere to ONVIF. Further considerations when building this type of system include the storage capacity of the NVR in relation to the number of cameras used, and ensuring that the power demand is covered for both the NVR and the intended number of cameras, when using mobile equipment.

The IP cameras were of the Linovision 4 K PoE IP underwater camera anti‐corrosion type, with a maximum depth of 165 ft, equipped with either 30 or 50 m cables (Figure A1a,b). These waterproof cameras send and retrieve information and power through a network cable. The cameras were mounted on foundations made of galvanized steel that had been filled with cast iron, either 5 or 8 kg, comprised of modified anchors (Figure A1a). When necessary, the network cables were weighed down to the river bottom by chains to prevent drifting in the stream and avoid snagging debris.

For this project, a mobile NVR (Hikvision AE‐MN7083 series) was used, which can connect up to 8 IP cameras via PoE interfaces (Figure A1c). The NVR connects to a DC‐12 V power source through an aviation plug (Figure A1d). The negative cord requires a 10A flat fuse, and the yellow accessory (ACC) wire should be connected to the positive red wire (Figure A1d). A male XT60 plug can be soldered to the wire ends for easier connection to the female XT60 of the battery (Figure A1e). At least one hard disk drive (HDD) needs to be mounted in its casing and locked in position for the NVR to power up and begin recording (Figure A1f). To activate and configure the mobile NVR, it can be connected to a computer via a network cable (Figure A1h).

A LiFePO4 (LFP) battery of 12 V and 100 AH was used to power the NVR and IP cameras (Figure A1i). The battery has DC‐12 V * 100AH = 1200 Watt‐hours. The manufacturer informed that each IP camera consumes 7 W, and the mobile NVR consumes 30 W. Hence, the setup should last for about 24 h with three cameras: 1200/(30 + (3 * 7)) = 23.6 h. However, during the study, the battery held for 48 h with three cameras. This may be because the NVR was set to record only during daylight, which was about 10 h a day in late October when the mobile NVR was used.

The battery was charged using an ISDT® 608 AC smart charger and connected to the charger through the XT60 connector (Figure A1j). The charger was configured as follows: Task—Charge; Chemistry—LiFe; Condition—3.6 V; Cells—4S; Current—8.0A. Select Start, then confirm the perform unbalancing task: LiFe‐4S. The battery charger beeps once the charging is complete. For the 12 V 100AH LFP battery, it takes approximately 12 h from empty to full.

The mobile NVR and IP cameras were activated by setting a password in the Hikvision® SADP software after connecting the mobile NVR to a laptop with a network cable. Once a password is set for the devices, their network parameters can be edited within the SADP software (Figure A2a). The default IP address of the mobile NVR is 192.168.1.64. The devices should share the same network, having IP addresses starting with 192.168.1.x, where x is any number between 1 and 254 that is not already assigned to another device in the network. The gateway of the mobile NVR and cameras was set to be identical: 192.168.1.1 (Figure A2a).

The IP address of the PC must be set to be on the same network as the mobile NVR. To set the IP address of a PC using Windows 10, follow these steps: Control Panel > Network and Sharing Centre > Change Adapter Settings > Ethernet > Internet Protocol Version 4 (TCP/IPv4). Here, the IP address can be set. For example, in the present study, 192.168.1.6 was used, the subnet mask was set to 255.255.255.0, and the default gateway to 192.168.1.1, which was the same gateway as the NVR and IP cameras. Then, click OK (Figure A2b). After this configuration, the mobile NVR can be accessed via a web browser by connecting the PC to the mobile NVR using a network cable (Figure A1h).

Once the PC, mobile NVR and cameras are on the same network, the mobile NVR can be configured by entering its IP address in a web browser (Figure A2c). To log in, use the username ‘admin’ and the password previously set in the SADP software. After logging in, set the mobile NVR's time and date by navigating to: Configuration > System Settings > Time Settings > Sync with Computer Time (Figure A2d). Format the HDD to enable recording by going to: Configuration > Storage Management > HDD Management > Format (Figure A2e). Set the recording schedule by going to: Configuration > Storage > Schedule Settings (Figure A2f).

Once the time is within the scheduled recording window, the devices will start recording. This can be indicated by the green light on the front of the NVR or by clicking on ‘Playback’ in the mobile NVR's web browser interface (Figure A1g). Note that the live view may not work in the version of Google Chrome used. However, you can obtain a live view from one camera at a time by navigating to: Configuration > Image and selecting the IP camera you want to view (Figure A2g).

To download videos from the HDD, go to ‘Playback’, choose the IP camera from which you want the videos, and press the download button. You will be prompted to set the time window for the files you want to download (Figure A2h).

With the default video settings of the mobile NVR (3840 * 2160 resolution and 20 frames per second), 2 h and 52 min of video took an average of 0.7645547 GB per hour (note: this is based on a small sample, so variations may occur). The mobile NVR accommodates 2 * 1 TB HDD, allowing for 2616 h of recording with one camera.

The stationary NVR, or simply NVR (model: NVR508P8‐Q2), draws power from a standard wall socket. This NVR comes with its own user interface and can be configured by connecting it to a monitor and a mouse (Figure A3a). Similar to the mobile NVR, the stationary NVR can be detected using the SADP software and configured via a web browser, as described for the mobile NVR. Therefore, no further description of the NVR's user interface is provided here.

On the back (Figure A3b), the NVR can connect up to eight IP cameras, interface with the web browser through a local area network (LAN), connect to a monitor via an HDMI port, and includes a USB port for actions such as downloading recorded videos via its user interface. The NVR508P8‐Q2 can accommodate two 8 TB HDDs (Figure A3c). Requiring 48 V DC, the NVR508P8‐Q2 needs a 100‐240 V power outlet, making it less mobile. However, the device can be set up in a tent or shed near the stream if there is a power outlet available (Figure A3d–f). Thanks to the 30 ‐or 50‐m‐long network cables, it was usually easy to find a suitable location for the cameras (Figure A3g–h).

With the default video settings of the NVR (3840 * 2160 resolution and 20 frames per second), 1 h of video took an average of 1.299198 GB (based on a 16‐h average). The mobile NVR accommodates 2 * 8 TB HDD, allowing for 12,315 h of recording with one camera.
